# Impact of COVID-19 pandemic on rates of congenital heart disease procedures among children: prospective cohort analyses of 26 270 procedures in 17 860 children using CVD-COVID-UK consortium record linkage data

**DOI:** 10.1136/openhrt-2024-003054

**Published:** 2025-03-25

**Authors:** Arun Karthikeyan Suseeladevi, Rachel Denholm, Sonya V Babu-Narayan, Shubhra Sinha, Serban Stoica, Tim Dong, Gianni D Angelini, Cathie L M Sudlow, Venexia Walker, Kate Brown, Massimo Caputo, Debbie A Lawlor, On behalf of the CVD-COVID-UK/COVID-IMPACT Consortium, Alastair Proudfoot

**Affiliations:** 1 Population Health Science, University of Bristol Medical School, Bristol, UK; 2 MRC Integrative Epidemiology Unit, University of Bristol Faculty of Health Sciences, Bristol, UK; 3 NIHR Bristol Biomedical Research Centre, University of Bristol, Bristol, UK; 4 Health Data Research UK South West, Bristol, UK; 5 British Heart Foundation, Health Data Research UK, London, UK; 6 National Heart and Lung Institute, Imperial College, London, UK; 7 Translational Health Sciences, University of Bristol, Bristol, UK; 8 Department of Surgery, University of Pennsylvania Perelman School of Medicine, Philadelphia, Pennsylvania, USA; 9 Great Ormond Street Hospital for Children NHS Trust, London, UK; 10 Institute of Cardiovascular Science, University College London, London, UK

**Keywords:** Electronic Health Records, COVID-19, Heart Defects, Congenital

## Abstract

**Background:**

The COVID-19 pandemic necessitated major reallocation of healthcare services. Our aim was to assess the impact on paediatric congenital heart disease (CHD) procedures during different pandemic periods compared with the prepandemic period, to inform appropriate responses to future major health services disruptions.

**Methods and results:**

We analysed 26 270 procedures from 17 860 children between 1 January 2018 and 31 March 2022 in England, linking them to primary/secondary care data. The study period included prepandemic and pandemic phases, with the latter including three restriction periods and corresponding relaxation periods. We compared procedure characteristics and outcomes between each pandemic period and the prepandemic period. There was a reduction in all procedures across all pandemic periods, with the largest reductions during the first, most severe restriction period (23 March 2020 to 23 June 2020), and the relaxation period following second restrictions (3 December 2020 to 4 January 2021) coinciding with winter pressures. During the first restrictions, median procedures per week dropped by 51 compared with the prepandemic period (80 vs 131 per week, p=4.98×10^−08^). Elective procedures drove these reductions, falling from 96 to 44 per week (p=1.89×10^−06^), while urgent (28 vs 27 per week, p=0.649) and life-saving/emergency procedures (7 vs 6 per week, p=0.198) remained unchanged. Cardiac surgery rates increased, and catheter-based procedure rates reduced during the pandemic. Procedures for children under 1 year were prioritised, especially during the first four pandemic periods. No evidence was found for differences in postprocedure complications (age-adjusted OR 1.1 (95% CI 0.9, 1.4)) or postprocedure mortality (age and case mix adjusted OR 0.9 (95% CI 0.6, 1.3)).

**Conclusions:**

Prioritisation of urgent, emergency and life-saving procedures during the pandemic, particularly in infants, did not impact paediatric CHD postprocedure complications or mortality. This information is valuable for future major health services disruptions, though longer-term follow-up of the effects of delaying elective surgery is needed.

**WHAT IS ALREADY KNOWN ON THIS TOPIC**

- Previous studies have shown that elective surgeries including congenital heart disease (CHD) procedures were actively postponed during the early days of the COVID-19 pandemic to reduce pressure on the healthcare system.

**WHAT THIS STUDY ADDS**

- This study quantifies the changes in CHD procedure volumes across different pandemic restriction phases in England, showing significant reductions in elective procedures during initial severe restrictions and winter pressures. The study did not observe increased postprocedure complications or 30-day mortality due to prioritisation strategies.

**HOW THIS STUDY MIGHT AFFECT RESEARCH, PRACTICE OR POLICY**

- The findings from this study can aid policy-makers in enhancing preparedness for future situations where healthcare delivery pressure may increase due to natural causes or diseases, such as climate change or pandemics.

## Introduction

 Infection with SARS-CoV-2 increases vascular permeability, damaging the respiratory system and causing long-term cardiovascular, renal, hepatobiliary and neurological effects.^1^,^12^ The COVID-19 pandemic strained healthcare resources, necessitating the postponement of specialist procedures like congenital heart disease (CHD) treatments to accommodate COVID-19 patients.^13^,^15^ Children born with CHD commonly require repeat cardiac catheterisation and surgical procedures (hereafter referred to as procedures) across childhood to ensure they maintain healthy cardiac structure and function as they grow.^16^,^18^ Several studies from different countries including China,^19^ India,^20^ Mexico,^21^ Turkey,^22^ Italy^23^ and the UK^24^ have explored the impact of the pandemic on procedures for children with CHD. These have compared the initial period, commonly the first 4–6 months, of the pandemic with a prepandemic period and report marked reductions in elective procedures. These have all been from selected regions or cities, with the number of procedures ranging from 29 to ~8000.^20 22^ None explored the effects of varying population restrictions over time, and few examined postprocedure complications and mortality.

Learning from the COVID-19 pandemic experiences is crucial for preparing for future disruptions to healthcare services, whether caused by other pandemics or factors such as extreme weather, wars or social disruptions like industrial action. Prioritising services for vulnerable populations during such disruptions is essential, while understanding their consequences is also necessary.

The aim of this study was to assess the impact of the COVID-19 pandemic on paediatric procedures for CHD in England. Specifically, we aimed to describe differences in overall, elective, urgent, emergency and life-saving procedures, and in postprocedure complications and mortality during various periods of pandemic restrictions and relaxations compared with the prepandemic period. We also explored whether the results varied by the child’s age, neighbourhood deprivation and ethnicity. Table 1 presents the different phases of pandemic restrictions and relaxations in England.

**Table 1:** Key restrictions during the different phases of the pandemic in England

Period (dates)	Policy on action	Description
Prepandemic (1 January 2018 to 22 March 2020)	None	
First restriction period(23 March 2020 to 23 June 2020)	Hospitality closure	Pubs, bars and restaurants must close but can operate a takeaway/delivery service
	Movement: stay at home	People are prohibited from leaving home without a reasonable excuse
	Gatherings: Social gatherings ban	Gatherings of more than two people are prohibited unless for a limited number of exempted purposes
	Movement: Staying away restriction	People are prohibited from staying away from home overnight without a reasonable excuse
	Movement: Outdoor recreation from 13 May	
	Gatherings: Rule of six (outdoors only) from 1 June	
	Hospitality: Non-essential retail opened on 15 June	
	Gathering: Support bubbles introduced on 15 June	
First relaxation (24 June 2020 to 4 November 2020)	Movement: Staying away restriction	People are prohibited from staying away from home overnight without a reasonable excuse
	Gatherings: Rule of six (outdoors) only	Gatherings of more than six people are prohibited unless they are for an exempted purpose. Exemptions include organised sports, small weddings and support groups
	Gatherings: Large gatherings banned from 1 July	Gatherings of more than thirty people are prohibited
	Hospitality: Opening times	Pubs, bars and restaurants must close at a specific time
	Gatherings: restored rule of six	
	Tier: introduced tier system (1, 2, 3)	
Second restrictions(5 November 2020 to 2 December 2020)	Hospitality: Business closure	Pubs, bars and restaurants must close but can operate a takeaway/delivery service
	Gatherings: Social gathering ban	Gatherings of more than two people are prohibited unless for a limited number of exempted purposes
	Movement: Staying at home—outdoor recreation allowed throughout	People are prohibited from leaving home without a reasonable excuse
Second relaxation(3 December 2020 to 5 January 2021)	Tier: reintroduced tier system, tier 4 introduced on 20 December	
Third restriction(6 January 2021 to 21 June 2021)	Hospitality: Business closure	Pubs, bars and restaurants must close but can operate a takeaway/delivery service
	Gatherings: Social gathering ban	Gatherings of more than two people are prohibited unless for a limited number of exempted purposes
	Movement: stay at home	People are prohibited from leaving home without a reasonable excuse
	Movement: Outdoor recreation allowed on 8 March Step 1 unlock	
	Step 1 unlock: children return to schools	
	Step1 unlock: Gatherings: Rule of six in outdoor reinstated	
	Movement: International travel ban	People are prohibited from leaving the United Kingdom without a reasonable excuse
	Hospitality: Opening times	Pubs, bars and restaurants must close at a specific time
	Step 2 unlock: Gatherings: reopening of outdoor attractions and settings	
	Step 3 unlock: not earlier than 17 May	
	Step 4 unlock: not earlier than 22 June	
Post third restrictions(22 June 2021 to 31 March 2022)	19 July: Most legal limits on social contact removed in England and final closed sectors of the economy reopened (eg, nightclubs)	
	14 September: PM unveils England’s winter plan for Covid-'Plan B' to be used if the NHS is coming under ‘unsustainable pressure’ and includes measures such as face masks	
	8 December: PM announces a move to ‘plan B’ measures in England following the spread of the Omicron variant	
	10 December: Face masks become compulsory in most public indoor venues under plan B	

## Methods

### Data sources

We used the National Congenital Heart Disease Audit database (NCHDA) as the central dataset. Established in 2000, the NCHDA evaluates outcomes of paediatric and congenital cardiovascular procedures in the UK. Data submission is mandatory for all centres performing these procedures, requiring information on diagnoses, procedures, urgency and outcomes up to 30 days postprocedure.^25^ The NCHDA data undergo validation tests for accuracy and completeness (online supplemental text).^26^,^28^

**Figure 1:**
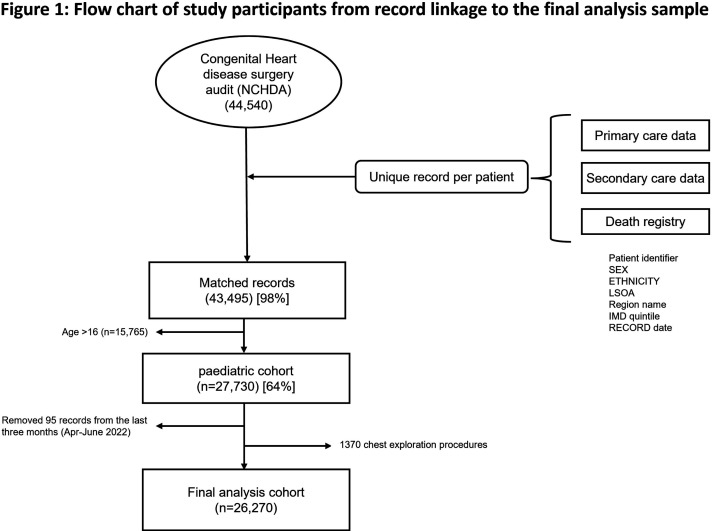
Flow chart of study participants from record linkage to the final analysis sample. IMD, Index of Multiple Deprivation; NCHDA, National Congenital Heart Disease Audit; LSOA, Lower Layer Super Output Areas.

NCHDA data were linked to electronic health records from General Practice Extraction Service Data for Pandemic Planning and research, Hospital Episode Statistics, and the Office of National Statistics death registry (figure 1; online supplemental text). Procedures performed between 1 January 2018 and 31 March 2022, among children under 16 in England were analysed.

The deidentified data were accessed within NHS England’s Secure Data Environment service^29^ via the BHF Data Science Centre's CVD-COVID-UK/COVID-IMPACT Consortium. Ethics and governance details are provided in the online supplemental text.

### Exposure

The exposure time periods reflect the UK’s COVID-19 responses (table 1).^30^

- Prepandemic (reference) period (1 January 2018 to 22 March 2020).
- First restriction period (23 March 2020 to 23 June 2020).
- First relaxation period (24 June 2020 to 4 November 2020).
- Second restriction period (5 November 2020 to 2 December 2020).
- Second relaxation period (3 December 2020 to 5 January 2021).
- Third restriction period (6 January 2021 to 21 June 2021).
- Third relaxation period (22 June 2021 to 31 March 2022).

### Outcomes

The key outcomes were procedure urgency status, postprocedure complications and postprocedure mortality. Urgency status classifies procedures as elective, urgent, emergency and life-saving. Postprocedure complications were defined as any operative or procedure complication occurring within 30 days after the procedure^25 26^ (see full list in online supplemental table S1). Postprocedure mortality was defined as deaths within 30 days of the procedure.^25 26^

### Covariates

Mortality following paediatric cardiac surgeries is compared across institutions using the Partial Risk Adjustment in Surgery 2 (PRAIS2) score.^31^ The PRAiS 2 score is estimated using factors such as activity group, specific procedure, primary diagnosis, ventricular physiology, child’s age and weight, and comorbidity, which are specific for cardiac surgeries.^2632^,^35^ To adjust for case mix in morbidity for all procedures included in this study, we used individual risk factors. Please refer to the online supplemental text for details on the derivation of the variables from the NCHDA dataset and the estimation of PRAiS 2 score, as well as online supplemental tables S2–S4 for the full list of primary diagnoses, specific procedures and risk factors used for adjustment.

### Analyses

The unit of analysis was each procedure, with children undergoing multiple procedures contributing more than once. To deal with the multilevel clustered data (ie, multiple procedures in children), robust SEs were used to calculate the 95%CI. We described the distribution of procedures and children’s sociodemographic characteristics using counts (%), median (IQR) and mean (SD).

We present the median (IQR) number of overall, elective, urgent and emergency/life-saving procedures per week for the prepandemic and pandemic periods, using the Wilcoxon rank sum test to compare differences between pandemic periods and the prepandemic period. Emergency and life-saving procedures were combined due to low numbers.

We used the Z-test to estimate the difference in mean percentage (95% CI) of procedures by (1) urgency: elective, urgent or emergency/life-saving, (2) type of procedure: cardiac surgery, intervention catheter or other and (3) age group: <1 year, 1 to <5 years, 5 to <10 years or 10 to <16 years.

We used age-adjusted logistic regression to estimate the ORs for (1) undergoing an urgent, emergency or life-saving procedure versus elective procedure, (2) postprocedure complications (yes vs no) and (3) postprocedure mortality within 30 days (yes vs no), comparing each pandemic period to the prepandemic period. For the mortality analysis, we additionally adjusted for case mix using PRAIS2 risk factors (online supplemental table S4).

### Sensitivity analyses

We assessed whether using individual PRAIS2 risk factors, rather than the weighted score, influenced our main results by comparing logistic regression outcomes for mortality with three adjustments: age only, age plus individual risk factors and age plus PRAIS2 score, specifically for cardiac surgeries.

### Exploratory subgroup analyses

We repeated the logistic regression analyses for subgroups based on age, ethnicity and deprivation quintiles, testing for statistical difference by including interaction terms between these variables and the pandemic periods. The online supplemental text provides justification and details on the characteristics adjusted for in the subgroup analyses.

### Dealing with missing data

No data were missing in the main analysis. Subgroup analyses for ethnicity (missing n=1,385 (5.3%)) and area deprivation (missing n=1405 (5.3%)) were limited to complete cases.

This analysis was performed according to a prespecified analysis plan published on GitHub, along with the phenotyping and analysis code (https://github.com/BHFDSC/CCU007_01).

## Results

The linkage of the NCHDA dataset with routine healthcare data was achieved for 43 495 (98%), with data from primary care, secondary care or ONS death registry data (91% linked to primary care, 99% to secondary care and 90% linked to both sources). After excluding the last low-reporting months (95 records from April to June 2022) and chest closure and exploration procedures (1370 records), the final analysis included 26 270 procedures performed on 17 860 children under 16 years of age, from 1 January 2018 to 31 March 2022 (figure 1 and table 2).

**Table 2:** Characteristics of children (<16 years) who underwent congenital heart disease surgical procedures in England between 1 January 2018 and 31 March 2022

Characteristics		N (%) of procedures	N (%) of children
		(N=26 270)	(N=17 860)
Age group, years	<1 year	8520 (32.4%)	5885 (33.0%)
	1 to <5 years	9235 (35.2%)	5730 (32.1%)
	5 to <10 years	4150 (15.8%)	2880 (16.1%)
	10 to 16 years	4365 (16.6%)	3365 (18.8%)
Gender	Male	14 290 (54.4%)	9475 (53.1%)
	Female	11 980 (45.6%)	8385 (46.9%)
Ethnicity (new)	White European	18 155 (69.1%)	12 370 (69.3%)
	South Asian	2555 (9.7%)	1680 (9.4%)
	African/Caribbean	1205 (4.6%)	860 (4.8%)
	Other	2975 (11.3%)	2020 11.3%)
	Missing	1385 (5.3%)	930 (5.2%)
Region	East Midlands	1770 (6.7%)	1165 (6.5%)
	East of England	1935 (7.4%)	1430 (8.0%)
	London	3400 (12.9%)	2470 (13.8%)
	North East	1125 (4.3%)	745 (4.2%)
	North West	3060 (11.6%)	1980 (11.1%)
	South East	3140 (12.0%)	2230 (12.5%)
	South West	1805 (6.9%)	1155 (6.5%)
	West Midlands	2910 (11.1%)	1885 (10.6%)
	Yorkshire and the Humber	2750 (10.5%)	1790 (10.0%)
	Missing	4380 (16.7%)	3010 (16.9%)
Index of Multiple Deprivation Quintiles	1 (most deprived)	7100 (27.0%)	4630 (25.9%)
	2	5520 (21.0%)	3695 (20.7%)
	3	4580 (17.4%)	3140 (17.6%)
	4	3955 (15.1%)	2750 (15.4%)
	5 (least deprived)	3715 (14.1%)	2720 (15.2%)
	Missing	1405 (5.3%)	930 (5.2%)
Primary diagnosis	Pulmonary atresia and stenosis*	2715 (10.3%)	
	Left ventricular outflow obstruction*	2625 (10.0%)	
	Patent ductus arteriosus	2255 (8.6%)	
	Arrhythmia	1845 (7.0%)	
	Ventricular septal defect	1845 (7.0%)	
	Misc. congenital primary diagnoses*	1825 (6.9%)	
	Transposition of great arteries*	1660 (6.3%)	
	Hypoplastic left heart syndrome	1640 (6.2%)	
	Fallot/DORV-Fallot type	1620 (6.2%)	
	Interatrial communication (‘ASD’)	1555 (5.9%)	
	Functionally univentricular heart	1500 (5.7%)	
	Atrioventricular septal defect	1400 (5.3%)	
	Primary atrioventricular valvar disease*	1240 (4.7%)	
	Acquired heart diseases	1115 (4.2%)	
	Misc. congenital terms	410 (1.6%)	
	Common arterial trunk (truncus arteriosus)	400 (1.5%)	
	Total anomalous pulmonary venous connection	235 (0.9%)	
	Missing†	385 (1.5%)	
Urgency of procedure	Elective	18 920 (72.0%)	
	Urgent	5815 (22.1%)	
	Emergency	1185 (4.5%)	
	Life-saving	300 (1.1%)	
	Missing	55 (0.2%)	
Procedure activity group	Cardiac surgery	12 955 (49.3%)	
	Interventional catheter	7250 (27.6%)	
	Diagnostic catheter	3045 (11.6%)	
	Electrophysiology	2295 (8.7%)	
	Mechanical support	720 (2.7%)	
	Missing	0 (0%)	
Complication‡	Yes (any)	2405 (9.2%)	
	ECMO	310 (1.2%)	
	Unplanned surgeries	380 (1.4%)	
	Necrotising enterocolitis	185 (0.7%)	
	Surgical site infection	75 (0.3%)	
	Pleural effusion	405 (1.5%)	
	Any other complication	1600 (6.1%)	
Discharge destination	Home	22 725 (86.5%)	
	Other hospital	2260 (8.6%)	
	Convalescence	20 (0.1%)	
	Death	550 (2.1%)	
	Death with referral to coroner	260 (1.0%)	
	Hospice/palliative care	35 (0.1%)	
	Other specialties in the same hospital	340 (1.3%)	
	Missing	80 (0.3%)	
Discharge status	Alive	25 395 (96.7%)	
	Died in hospital	815 (3.1%)	
	Missing	55 (0.2%)	
Hospital stay	Duration of hospitalisation (median (IQR) days	5 (1–11)	

* Smaller groups were combined to form larger groups (Ffull information in Supplementary Table 1online supplemental table S1).

† Records with diagnostic codes other than NCHDA approved diagnostic codes were assigned as missing.

‡ No information on missing data as the field is completed only in relevant cases. All counts below 10 are suppressed and others are rounded to the nearest multiple of 5 as per the safe output services guidelines of NHS England’s Secure Data Environment service for England.^45^

ECMOExtra Corporeal Membrane OxygenationNCHDANational Congenital Heart Disease Audit databaseNHSNational Health Service

Table 2 presents the distributions of sociodemographic and clinical characteristics for all procedures throughout the analysis period. The predominant ethnic group was white European, and the London region had the highest proportion of cases. Pulmonary atresia and stenosis and left ventricular outflow obstruction were the most common primary diagnosis, while total anomalous pulmonary venous connection was the least common. Of all the procedures, 72% (n=18 920) were elective, with the most being cardiac surgeries (n=12 955, 49%) or intervention catheters (n=7250, 28%). During the study period, postprocedure complications were below 10% and 2.1% children died within 30 days.

### Reduction in the median (IQR) number of overall procedures per week during pandemic periods compared to the prepandemic period

**Figure 2:**
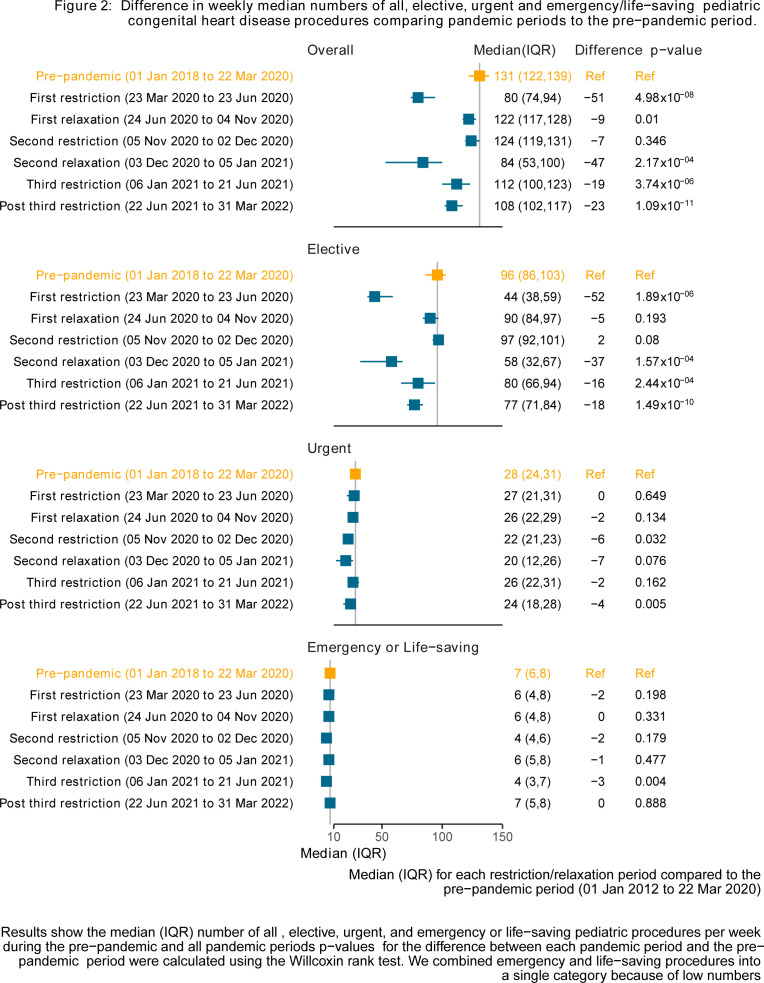
The difference is in the weekly median numbers of all, elective, urgent and emergency/life-saving paediatric congenital heart disease procedures comparing pandemic periods to the prepandemic period. Median (IQR) for each restriction/relaxation period compared to the prepandemic period (1 January 2018 to 22 March 2020). Results show the median (IQR) number of all, elective, urgent and emergency or life-saving paediatric procedures per week during the prepandemic and all pandemic periods. P values for the difference between each pandemic period and the prepandemic period were calculated using the Wilcoxon rank test. We combined emergency and life-saving procedures into a single category because of low numbers.

The largest declines occurred during the first and most severe pandemic restriction and the relaxation following the second restriction (figure 2). Elective procedures drove these reductions, decreasing from 96 per week to 44 per week during the first restriction (p=1.89×10^−06^). Urgent procedures showed no change (27 vs 28 per week, p=0.649) nor did life-saving/emergency procedures (6 vs 7 per week, p=0.198). Differences in mean percentage of urgent and emergency/life-saving procedures between pandemic and prepandemic period followed similar patterns (online supplemental figure S1).

### Difference in mean percentage of procedure types performed during pandemic periods compared with the prepandemic period

**Figure 3:**
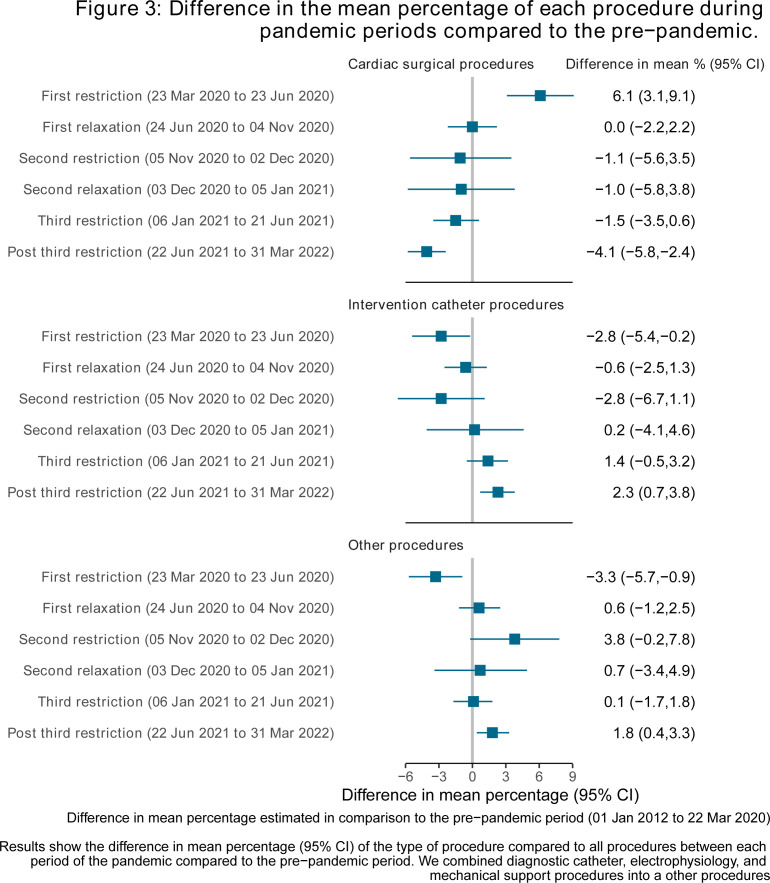
Difference in the mean percentage of each procedure during pandemic periods compared to the prepandemic. Difference in mean percentage estimated in comparison to the prepandemic period (1 January 2018 to 22 March 2020). Results show the difference in mean percentage (95% CI) of the type of procedure compared to all procedures between each period of the pandemic compared to the prepandemic period. We combined diagnostic catheter, electrophysiology and mechanical support procedures into other procedures.

During the first restriction, there was a 6.1% (95% CI: 3.1%, 9.1%) increase in cardiac surgeries, accompanied by reduction in catheter (−2.8% (95% CI: −5.4, –0.2)) and other (−3.3% (95% CI: −5.7, 0.9)) procedures (figure 3). This was followed by a gradual return towards prepandemic levels until the final pandemic periods, with reduction in cardiac surgeries and increase in catheter procedures compared with prepandemic levels. Online supplemental table S7 details the differences in mean percentages for each specific procedure. Among the 86 specific procedures, 36 were less likely, 46 were more likely and 6 showed no difference between the first restriction period and prepandemic levels. Procedures that were less likely included electrophysiological ablation, atrial septal defect, atrial septal defect transluminal, total cavo-pulmonary connection (known as Fontan’s procedure) and patent ductus arteriosus transluminal, while those that were more likely included Fallot’s, balloon atrial septostomy, coarctation hypoplasia, superior vena cava to pulmonary artery anastomosis (known as Glenn’s anastomosis). There was no strong evidence of differences in specific procedures during other pandemic periods, though we had limited power at this granular level.

### Age and difference in mean percentage of procedures during pandemic periods compared with the prepandemic period

**Figure 4:**
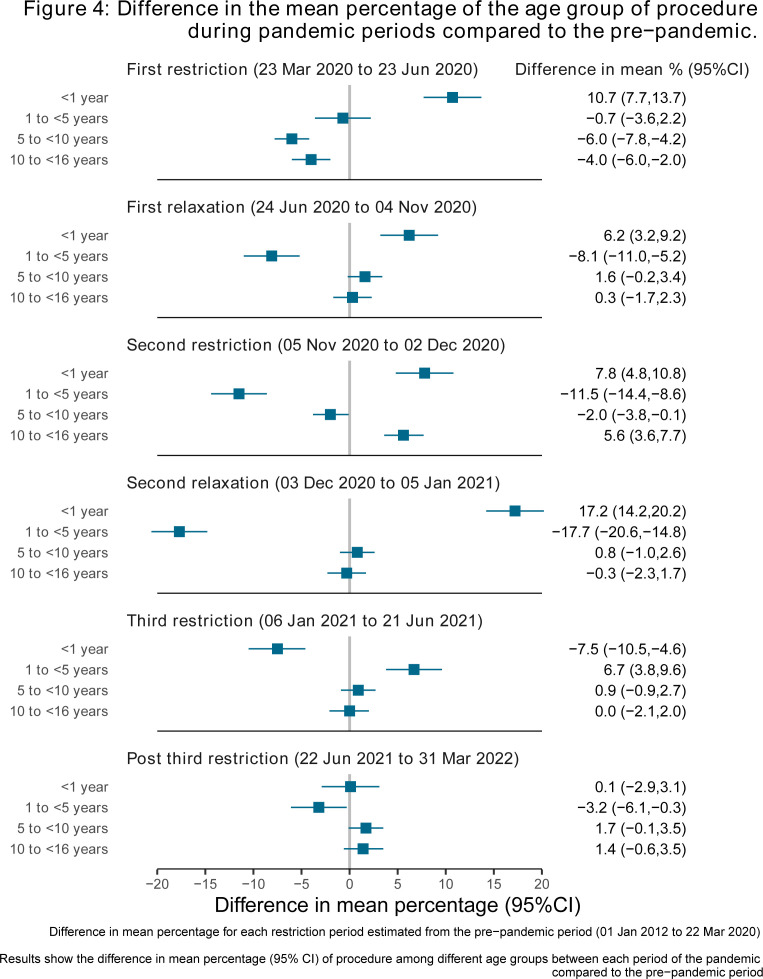
Difference in the mean percentage of the age group of procedure during pandemic periods compared to the prepandemic. Difference in mean percentage for each restriction period estimated from the prepandemic period (1 January 2018 to 22 March 2020). Results show the difference in mean percentage (95% CI) of procedure among different age groups between each period of the pandemic compared to the prepandemic period.

Across all pandemic periods, except the third restriction and postpandemic period, procedures among children under 1 year were higher than prepandemic levels (figure 4). In the third restriction period, procedures in this age group were lower than in the prepandemic period. For other age groups, patterns varied across the pandemic periods. By the postpandemic period, procedures for children aged 1 to below 5 were lower than the prepandemic period, while the other three age groups remained similar to the prepandemic period.

### Logistic regression

**Figure 5:**
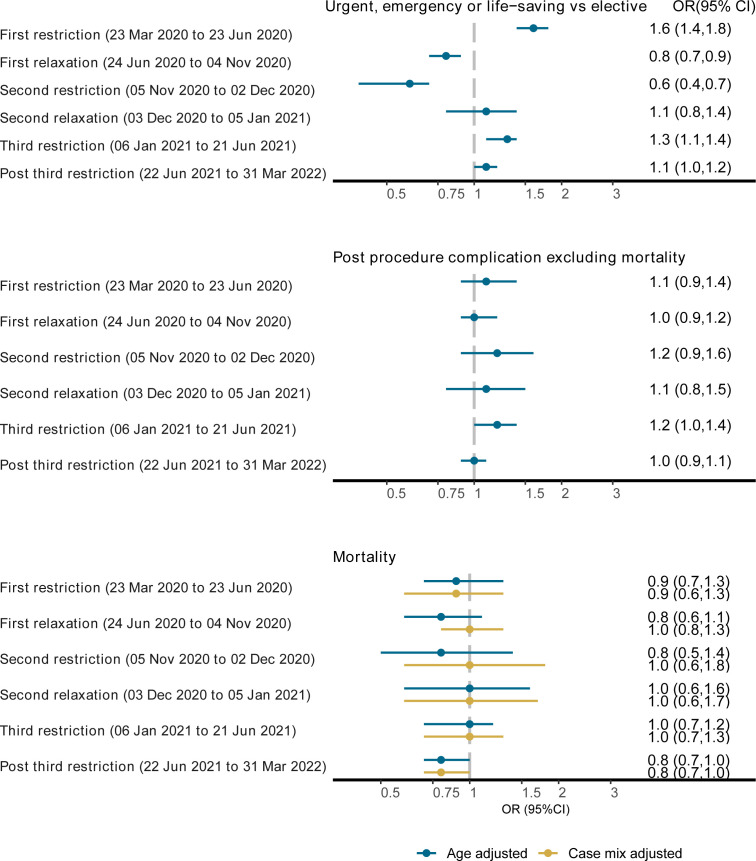
ORs of urgency, postprocedure complications and mortality within 30 days of a procedure comparing pandemic periods to the prepandemic period. OR (95% CI) estimated in comparison to the prepandemic period (1 January 2018 to 22 March 2020). Results show the age-adjusted ORs of urgent/emergency/life-saving procedure versus elective, postprocedure complications (yes vs no) and age, and age plus case mix adjusted odds of mortality within 30 days of a procedure (yes vs no) during different periods of the pandemic compared with the prepandemic period. We combined urgent, emergency and life-saving procedures into a single category.

There was a marked increase in odds of urgent, emergency or life-saving procedures in the first period of restrictions (age-adjusted OR 1.6 (95% CI: 1.4, 1.8)), followed by a reduction in the subsequent relaxation period (age-adjusted OR 0.8 (95% CI: 0.7, 1.9)). We did not find evidence of differences in postprocedure complications or postprocedure mortality within 30 days during any pandemic period, compared with prepandemic (figure 5).

### Sensitivity analysis

We found no difference in the odds of mortality within 30 days, compared with prepandemic, in age-adjusted, age plus individual case mix risk factor adjusted and age plus PRAIS2 risk score adjusted models (online supplemental figure S2).

### Exploratory subgroup analyses

There were statistical differences in the association of pandemic periods with the procedure urgency across age groups (interaction p=2.96×10^−09^; online supplemental figure S3). The odds of urgency increased during the first restriction period for all age groups, especially for older children, but this effect diminished in the subsequent periods. There was no evidence of differences by age group for the odds of postprocedure complications (interaction p=0.09; online supplemental figure S4) and imprecise estimates with wide confidence intervals precluded strong conclusions about mortality (interaction p=0.007; online supplemental figure S5).

Ethnic group analysis showed increased odds for urgency except for South Asian children during the first restriction period, with patterns returning to prepandemic rates by the third relaxation period (interaction p=0.003 (online supplemental figure S6). There were no differences in the association of pandemic periods with complications by ethnicity (interaction p=0.580; online supplemental figure S7). Although there was statistical evidence of differences in postprocedure mortality between ethnic groups (interaction p=4.7×10^−05^ (online supplemental figure S8), estimates were too imprecise for meaningful conclusions.

Analysis by residential area deprivation showed no evidence that associations of pandemic periods with urgent, emergency or life-saving procedures (interaction p=0.744; online supplemental figure S9) or complications (interaction p=0.6367; online supplemental figure S10). Estimates for mortality were too imprecise for robust conclusions (interaction p=1.03×10^−05^; online supplemental figure S11).

## Discussion

This study is, to our knowledge, the largest study using whole population data to examine the impact of the COVID-19 pandemic response on CHD procedures in children. We found that the median number of CHD procedures per week was lower during all pandemic periods compared with prepandemic levels. The largest reductions occurred during the first, most severe restrictions and the relaxation period following the second restrictions, coinciding with winter pressures. These reductions were primarily driven by reductions in elective procedures, while urgent and emergency/life-saving procedures remained stable compared with prepandemic rates. There was evidence of prioritising cardiac surgery over catheterisation and prioritising infants during the pandemic. Reassuringly, we found limited evidence of increased postprocedure complications or mortality during the pandemic compared with the prepandemic levels.

Children with complex CHD require repeat procedures and/or percutaneous/hybrid interventions throughout their lives.^17 18^ Some conditions, such as transposition of the great arteries and hypoplastic left heart syndrome, are time-sensitive and require immediate perinatal attention. The prioritisation of urgent, emergency and life-saving CHD procedures over elective ones, as seen in our and other studies,^14 15 36^ may explain why we observed no differences in postprocedure complications or mortality within 30 days. The paediatric and congenital cardiac services programme in China,^19^ Brazil,^37^,^39^ India ^20^and Lithuania^40^ reported similar decrease in volume and increase in complexities of surgeries during the early days of the pandemic. Similar to our study, the prioritisation of urgent cases and those among younger age groups was observed in the Lithuanian^40^ and Indian^20^ studies. Consistent with our findings, there was no increase in mortality in the Brazilian study. The Indian paediatric cardiac services saw an increase in overall in-hospital and postoperative mortality during 2020 when compared with 2019.^20 37^ Whether there were increases in paediatric procedures for CHD specifically is unclear. The Lithuanian programme saw the length of stay in hospital per procedure increase during the pandemic, but it is unclear whether that was due to increased complications or other factors.

Overall, there is consistency across different studies in relation to reductions in paediatric CHD procedures, with prioritisation of emergency procedures and younger children. In our UK study, the largest to date, and the only one to explore different phases of the pandemic, we saw no related increase in postoperative complications. The higher mortality in India for all procedures in 2020 compared with 2019 and of longer stay in hospital for all procedures in Lithuania may not be driven specifically by the pandemic and changes to healthcare as a result of that, and may be influenced by different healthcare systems between countries.

While our results are reassuring, the impact of delays in elective surgery and the broader effects of major disruptions to specialised surgery care during the pandemic—such as resource reallocation, staff fatigue, illness and family anxiety—remain unknown. Continuing this study over a longer period will allow us to explore the pandemic’s impact on children’s cardiovascular and overall health. New linkages to educational administrative datasets and family members’ healthcare records will facilitate investigations into effects on children’s educational outcomes and the mental health of children, parents and other family members.

We explored whether the associations we observed differed by the child’s age, ethnicity and residential area deprivation and found statistical evidence for some. The increased odds of urgent, emergency or life-saving procedures in older children during the first restriction period and other pandemic periods likely reflect the prioritisation of procedures in younger children. This indicates that infants were more likely to have elective, urgent, emergency or life-saving procedures, compared with older children. However, we acknowledge that our subgroup analyses were underpowered and like all subgroup analyses, require replication.

### Strengths and limitations

A key strength of this study is the use of country-wide data for all the CHD procedures performed in England. This is made possible by the mandatory requirement for all institutions conducting paediatric cardiac procedures to submit complete data to NCHDA. We linked this data to primary and secondary care records to conduct our analyses. The NCHDA ensures high accuracy through rigorous validation processes, including complication and mortality verification. With a Data Quality Index score >90% considered good, all paediatric centres met this standard in the recent audit report.^28 41^ Furthermore, for our main analyses, there were no missing data. To our knowledge, this is the largest study to date, allowing us to examine how healthcare provision for paediatric CHD procedures changed over an extended period of varying restrictions. While the large numbers enabled exploratory subgroup analyses, we recognise that even with substantial data, estimates remain imprecise, and larger studies would be necessary for more robust conclusions. There were small amounts of missing data for ethnicity and residential area deprivation (5.3% each), which could bias results if concentrated in specific subgroups. This is not possible to explore. However, since these data come from electronic health records and the missing proportion is small, we suspect any bias would be minimal.

Between-hospital variation in the timing of mandatory data uploads can lead to incomplete data or artificial trends towards the most recent months of analysis. To mitigate this, we initially extracted data until 30 June 2022 but excluded the last 3 months, including data up to 31 March 2022.

Our analysis operates at a population level, limiting our ability to map individual patient experiences or quantify differences in delays, particularly regarding the impact of elective surgery delays. We categorised the pandemic months into six periods of restrictions and relaxation; however, these restrictions were not uniformly applied (see table 1). For instance, the first period was the most stringent and consistent nationwide, while the second involved some regional variations in restrictions, and the third included six gradual steps of easing measures until the pandemic was declared over. We a priori decided to analyse each period of any restrictions in the same way to increase power to detect differences, including for the rarer outcomes of postprocedure complications and postprocedure mortality. Thus, our results cannot be interpreted as potential effects of specific restrictions; rather, they illustrate the broader impact of health services pressures that necessitate delaying elective procedures and prioritising more urgent cases.

### Implications and conclusions

Our results suggest that when pressures on health services result in prioritisation of urgent, emergency and life-saving procedures in children with CHD and delaying elective procedures, this does not result in increased postprocedure complications or mortality over a period of 2 years. These findings have implications for future health service provision, particularly during infectious disease epidemics or global pandemics as well as during extreme weather events common across Europe.^42^,^44^ Notably, during the relaxation period following the second restriction, the median rates of overall and elective procedures dropped to levels comparable to those in the first restriction period, exceeding the reductions seen during the second restriction. This second relaxation occurred during winter (3 December 2020 to 5 January 2021) and may reflect winter pressures. As climate change intensifies the frequency of weather extremes, such pressures are likely to rise, highlighting the need for strategies to mitigate climate change and effective plans to manage health services pressures from various sources.

In conclusion, our findings suggest that delaying elective procedures in children with CHD to prioritise urgent, emergency and life-saving procedures does not increase procedure-related complications or 30-day mortality, making this approach appropriate in times of healthcare pressures. However, further research is essential to assess the long-term effects of such delays on cardiovascular health of children and the mental health and well-being of affected children, their parents and family members.

## supplementary material

10.1136/openhrt-2024-003054 online supplemental file 1

## Data Availability

Data may be obtained from a third party and are not publicly available.
